# Heterogeneity of SARS-CoV-2 virus produced in cell culture revealed by shotgun proteomics and supported by genome sequencing

**DOI:** 10.1007/s00216-021-03401-9

**Published:** 2021-05-20

**Authors:** Fabrice Gallais, Olivier Pible, Jean-Charles Gaillard, Stéphanie Debroas, Hélène Batina, Sylvie Ruat, Florian Sandron, Damien Delafoy, Zuzana Gerber, Robert Olaso, Fabienne Gas, Laurent Bellanger, Jean-François Deleuze, Lucia Grenga, Jean Armengaud

**Affiliations:** 1 Département Médicaments et Technologies pour la Santé (DMTS), Université Paris-Saclay, CEA, INRAE SPI, 30200 Bagnols-sur-Cèze, France; 2 Centre National de Recherche en Génomique Humaine (CNRGH), Institut de Biologie François Jacob, CEA, Université Paris-Saclay, 91057 Evry, France

**Keywords:** SARS-CoV-2, Viral particles, Mutations, Variants, Mass spectrometry, Genome sequencing

## Abstract

COVID-19 is the most disturbing pandemic of the past hundred years. Its causative agent, the SARS-CoV-2 virus, has been the subject of an unprecedented investigation to characterize its molecular structure and intimate functioning. While markers for its detection have been proposed and several diagnostic methodologies developed, its propensity to evolve and evade diagnostic tools and the immune response is of great concern. The recent spread of new variants with increased infectivity requires even more attention. Here, we document how shotgun proteomics can be useful for rapidly monitoring the evolution of the SARS-CoV-2 virus. We evaluated the heterogeneity of purified SARS-CoV-2 virus obtained after culturing in the Vero E6 cell line. We found that cell culture induces significant changes that are translated at the protein level, such changes being detectable by tandem mass spectrometry. Production of viral particles requires careful quality control which can be easily performed by shotgun proteomics. Although considered relatively stable so far, the SARS-CoV-2 genome turns out to be prone to frequent variations. Therefore, the sequencing of SARS-CoV-2 variants from patients reporting only the consensus genome after its amplification would deserve more attention and could benefit from more in-depth analysis of low level but crystal-clear signals, as well as complementary and rapid analysis by shotgun proteomics.

**Graphical abstract:**

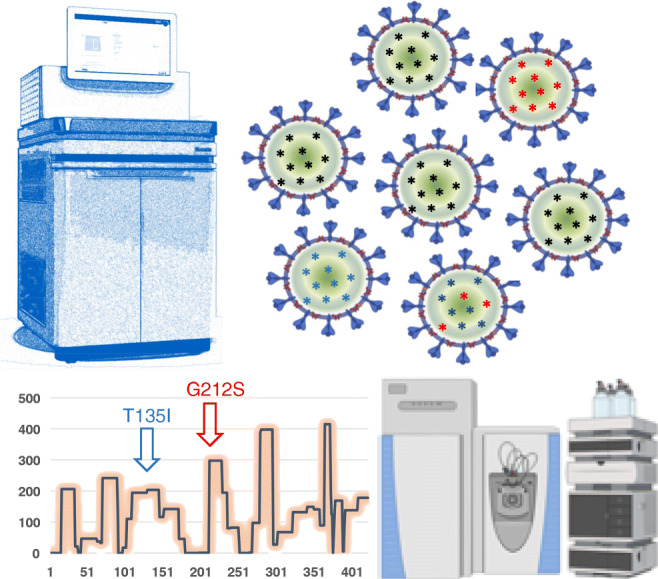

**Supplementary Information:**

The online version contains supplementary material available at 10.1007/s00216-021-03401-9.

## Introduction

Severe acute respiratory syndrome coronavirus-2 (SARS CoV-2) belongs to the genus *Betacoronavirus* (lineage B), subgenus *Sarbecovirus*. It was first described in December 2019 as the virus responsible for the current global COVID-19 pandemic [1]. The ≈30-kb single-stranded RNA genome of SARS-CoV-2 encodes for 4 structural proteins assembled into viral particles and 25 additional proteins required to hijack the molecular machinery of the host and produce copies of the viral RNA and the infective virions [2]. The four structural proteins are the spike (S), membrane (M), envelope (E), and nucleocapsid (N) proteins. These proteins comprise 1273, 222, 75, and 419 amino acids, respectively. The S protein is a glycoprotein that plays a pivotal role in viral attachment, fusion, and entry into host cells [3]. The precursor protein is cleaved into two subunits, an N-terminal S1 subunit responsible for the attachment to the host and a C-terminal S2 subunit mediating membrane fusion. Three S1/S2 heterodimers assemble to form a trimer spike protruding from the viral envelope. The membrane (or Matrix) protein is a glycoprotein with a central role in virion assembly and morphogenesis. The envelope (E) protein is a small membrane protein with a single alpha-helical transmembrane domain which plays a central role not only in virus morphogenesis and assembly, but also in pathogenesis during COVID-19 infection as it acts as a viroporin by forming homopentameric protein-lipid pores in the host membrane. The nucleocapsid (N protein-RNA complex) protects and organizes the RNA molecule into the viral lumen. Interestingly, structural studies of viral particles revealed an extensive heterogeneity of its molecular architecture [1].

Whole SARS-CoV-2 genome sequencing of numerous samples identified different lineages and sub-lineages along with the progressive spread of the virus over the planet and its propensity to evolve by accumulative mutations. Protein structural models highlighted that most substitutions map to protein surfaces and some could help the virus to evade the host immune system response [4]. The specific drastic countermeasures to limit the spread of the virus worldwide such as massive lockdown and COVID-19 shielding measures have increased the selective pressure on the virus. The emergence of new variants with an evident selective advantage such as the B.1.1.7 lineage in England triggers a new phase of the pandemic during autumn 2020 [5]. This lineage possesses not only a large number of non-synonymous substitutions, some being at key locations of the S protein, but also a short deletion that has been the cause of the S-gene target failure in at least one diagnostic PCR test (i.e. the Thermo Fisher TaqPath COVID-19 assay). The appearance of the B.1.351 lineage in South Africa, P.1 variant in Brazil, B.1.617 variant in India, and many others to come that could have major impacts on the course of the pandemic was somehow expected [6, 7]. Furthermore, medical countermeasures such as the use of convalescent plasma to treat patients or the massive vaccination campaign could result in the appearance of new evading variants. Indeed, marked intra-host genomic evolution of SARS-CoV-2 was observed in immunocompromised patients suffering long-term COVID-19 and treated with convalescent plasma [8].

Proteomics has been used to characterize vaccine components as exemplified with the contagious caprine pleuropneumonia vaccines [9] and to monitor the manufacturing process of live attenuated virus vaccines upstream and during purification stages [10]. We have also recently shown how shotgun proteomics of SARS-CoV-2 infected cells can be of help to optimize whole viral particle antigen production for vaccines [11]. Proteomics has been shown quite useful to understand the molecular mechanisms deployed by the virus to hijack the host machinery and to define relevant markers for diagnostics [1]. Interestingly, RNAseq was used to determine the transcriptome of SARS-CoV-2 grown in Vero E6 cells, a cell line widely used to propagate the virus, and evidenced an in-frame deletion of the gene encoding the Spike glycoprotein [12]. This deletion was confirmed at the protein level by tandem mass analysis of the infected cells.

Here, we explored the heterogeneity of SARS-CoV-2 viral particles produced and purified from Vero E6 cells to be used as standards in different virology assays. We carried out shotgun proteomics on these purified particles and interpret the MS/MS spectra with various strategies to detect the presence of possible variant peptides arising from mutations. We documented that cell cultivation to produce virus particles can induce significant changes at the structural protein level. We reported that tandem mass spectrometry is a quick and valuable approach to evaluate this heterogeneity.

## Materials and methods

### Virus production, purification, and inactivation

Vero E6 cells (Vero 76 – clone E6, ATCC CLR-1586, American Type Culture Collection) seeded at 1 × 10^7^ into 150-cm^2^ flasks were grown to cell subconfluence in 25 mL of Dulbecco’s modified Eagle’s medium (DMEM, Gibco, Thermo Fisher) supplemented with 5% of foetal calf serum (FCS, HyClone Fetal bovine serum, Cytiva) and 0.5% penicillin-streptomycin (Gibco) for one night at 37 °C under 9% CO_2_. Cells were infected at a multiplicity of infection (MOI) of 0.001 with SARS-CoV-2 strain 2019-nCoV/Italy-INMI1 (Genbank MT066156) which was provided by the Lazzaro Spallanzani National Institute of Infectious Diseases (Rome, Italy) via the EVAg network (European Virus Archive goes global). SARS-CoV-2 virions were purified after a total of four passages, as the virus was received as a stock derived from two consecutive passages and a pre-amplification was performed in our facility on reception. Viruses were harvested at 3 days post-infection (dpi). The viral suspension was recovered after centrifugation at 2500 rpm for 10 min performed to remove cell debris. Aliquots of 33 mL of the viral suspension were laid on 5 mL of 20% (w/v) sucrose cushions prepared in NaCl 0.1 M, EDTA 1 mM, and 10 mM Tris HCl buffer (pH 7.4) (TNE buffer) in Ultra-Clear 38-mL tubes (Beckman Coulter). Samples were centrifuged at 25,000 rpm for 2 h at 4 °C in a Beckman SW32 rotor. Supernatants were discarded and pellets were solubilized in 150 μL of cold TNE buffer. Aliquots of 1.5 mL of this sample were laid on a five-step 20–60% (w/v) sucrose gradient prepared in Ultra-Clear 13-mL tubes (Beckman Coulter). The tubes were centrifuged at 35,000 rpm for 2 h at 4 °C in a Beckman SW41 rotor. After recovery of the virus opalescent bands, an aliquot of 1 mL of the viral suspension was heat-inactivated for 1 h at 75 °C while the remaining sample was stored at −80 °C. Plaque assay titration was used to quantify the purified virus and to validate the viral inactivation. Experiments performed with live SARS-CoV-2 samples were conducted in a level 3 biosafety facility.

### Evaluation of virus infectivity

Vero E6 cells were seeded into 12-well culture plates at a density of 0.25 × 10^6^ cells/well in 2 mL of DMEM supplemented with 5% FCS and 0.5% penicillin-streptomycin and incubated overnight at 37 °C under 9% CO_2_. Tenfold dilutions of virus stock in DMEM supplemented with 2.5% FCS and 0.5% penicillin-streptomycin (500 μL of volume) were added to each well. The virus was then allowed to attach to the cells for 1 h at 37 °C. An overlay consisting of a semi-solid medium was prepared during the viral adsorption with 4% carboxymethylcellulose (Calbiochem) mixed in a 1:1 ratio with DMEM supplemented with 10% FCS and 0.5% penicillin-streptomycin. Two millilitres of DMEM:carboxymethylcellulose mixture was added to each well and plates were incubated at 37 °C under 5% CO_2_ for 3 days. On day 3, the overlays were gently removed and 500 μL of 0.2% (w/v) crystal violet (Sigma-Aldrich) prepared in 20% ethanol/3.7% formaldehyde (Sigma-Aldrich) was added to each well. After a 25-min incubation, the crystal violet solution was removed with a pipette and the well was washed with 2 mL phosphate buffer saline pH 7.4. This wash was repeated until excess crystal violet was removed. The lyzed plaques were counted at the dilution where 10–100 plaques were visualized. The titre in plaque forming unit (PFU) was expressed per volume of the initial sample.

### Proteolysis of inactivated viral particles

A volume of 60 μL of inactivated viral particles sample containing 0.614 μg/μL of proteins was diluted with 20 μL of LDS1X (Invitrogen). The sample was heated at 99 °C for 5 min. A volume of 25 μL (11.5 μg) of this sample was loaded onto a NuPAGE 4–12% Bis-Tris gel. Proteins were subjected to a short (5 min) SDS-PAGE migration. The polyacrylamide gel was then stained with Coomassie SimplyBlue SafeStain (Thermo Fisher) for 5 min and then destained overnight with distilled water. The proteins were excised from the gel as a single polyacrylamide small band, reduced with 25 mM of dithiothreithol (Sigma-Aldrich) and treated with iodoacetamide (Sigma-Aldrich), and then subjected to in-gel trypsin proteolysis with Trypsin Gold (Promega) at a ratio of enzyme to protein of 2% and in the presence of 0.01% ProteaseMAX surfactant (Promega) following the procedure described by Hartmann et al. (2014). The peptide fraction obtained (50 μL) was analysed directly by tandem mass spectrometry.

### Tandem mass spectrometry

The peptides were analysed with a Q-Exactive HF tandem mass spectrometer (Thermo Fisher) coupled with an UltiMate 3000 nanoRSLC system (Dionex-LC). The instrument was operated in a data-dependent mode. A volume of 10 μL of peptides (out of the 50 μL obtained after trypsin proteolysis) was injected onto an Acclaim PepMap100 C18 precolumn (5 μm, 100 Å, 300 μm id × 5 mm) for desalting, and then resolved on a nanoscale Acclaim PepMap 100 C18 column (3 μm, 100 Å, 75 μm id × 50 cm) with a 60-min gradient of acetonitrile. The flow rate was set at 0.2 μL/min and the gradient was developed in 50 min from 4 to 25% of 80%CH_3_CN/20%H_2_O in the presence of 0.1% formic acid and then 10 min from 25 to 40%. Full scan of peptide ions in the ultra-high-field Orbitrap analyser was done from *m/z* 350 to 1500 at a resolution of 60,000. MS/MS fragmentation was done sequentially on the 20 most intense ion signals (Top20 method) but selecting only positive charges 2+ or 3+, an intensity threshold of 50,000, and with a dynamic exclusion of 10 s. MS/MS spectra were acquired at a resolution of 15,000. Three analytical replicates were acquired after insertion of a new pre-column, validation of the chromatographic system with injections of Cytochrome C, certification of the performances of the mass spectrometer with HeLa cell extract, calibration, and injection of a blank sample, a procedure that prevented cross-contamination with other samples.

### Proteomic data interpretation

MS/MS spectra were assigned to peptide sequences using the Mascot 2.5.1 search engine (Matrix Science). First, a database comprising the Italy-INMI1 SARS-CoV-2 protein sequences (ISL_410545), the UniProt *Chlorocebus* sequences, and 23 *Bos taurus* sequences corresponding to the most abundant proteins from FCS present in the cell culture medium [13] was created. This database comprises 27,696 protein sequences, totalling 13,824,673 amino acids. A second database comprising all possible variants for SARS-CoV-2, the UniProt *Chlorocebus* sequences, and the 23 *Bos taurus* sequences was designed. This second database contains 30,057 protein entries totalling 19,833,126 residues. Peptide-to-MS/MS spectrum assignment was done with full trypsin specificity, maximum of two missed cleavages, static modification of cysteine into carbamidomethylated cysteine, and variable oxidation of methionine, and deamidation of asparagine and glutamine. Mass tolerances were set at 5 ppm and 0.02 Da on the parent ion and the MS/MS fragment, respectively. Only peptide matches presenting a Mascot peptide score with a *p* value lower than 0.05 were retained. Proteins were validated with a false discovery rate below 1% and their abundances were established in each sample based on their spectral counts (SC). An additional search with Mascot was performed in error-tolerant mode.

### Nucleic acid extraction, qPCR, and whole-genome sequencing

SARS-CoV-2 RNA from the purified viral particles was extracted using the NucleoSpin RNA Virus Mini kit (Macherey-Nagel) according to the manufacturer’s instructions. The number of RNA copies was evaluated by RT-qPCR using the SuperScript III Platinum One-Step RT-qPCR Kit (Thermo Fisher) and a CFX96 Touch Real-Time PCR Detection System Thermal Cycler (BioRad) as previously described [11]. CleanPlex SARS-CoV-2 kit (Paragon Genomics, Inc., Hayward, CA, USA) was used following the manufacturer’s protocol for sequencing libraries preparation, based on amplicon-based target enrichment. Reverse transcription was performed using 100 ng of RNA samples. Then, multiplex PCR reactions were performed using the CleanPlex SARS-CoV-2 panel. In total, 343 pairs of primers separated into two pools covering the entire genome of SARS-CoV-2 were used with amplicons ranging from 116 to 196 bp, with a median size of 149 bp. The libraries were quantified using Qubit 2.0 fluorometer (Invitrogen) and fragment sizes were analysed in the LabChip GX system (Perkin, USA). Pooled libraries were sequenced on a NovaSeq 6000 (Illumina), with SP, 2×150bp flowcell.

### Data repository

The mass spectrometry and proteomics datasets are available through the ProteomeXchange Consortium via the PRIDE partner repository (https://www.ebi.ac.uk/pride/), under dataset identifiers PXD025131 and 10.6019/PXD025131 for direct interpretation and PXD025130 and 10.6019/PXD025130 for error-tolerant interpretation.

## Results

### Production and purification of SARS-CoV-2 viruses

**Fig. 1:**
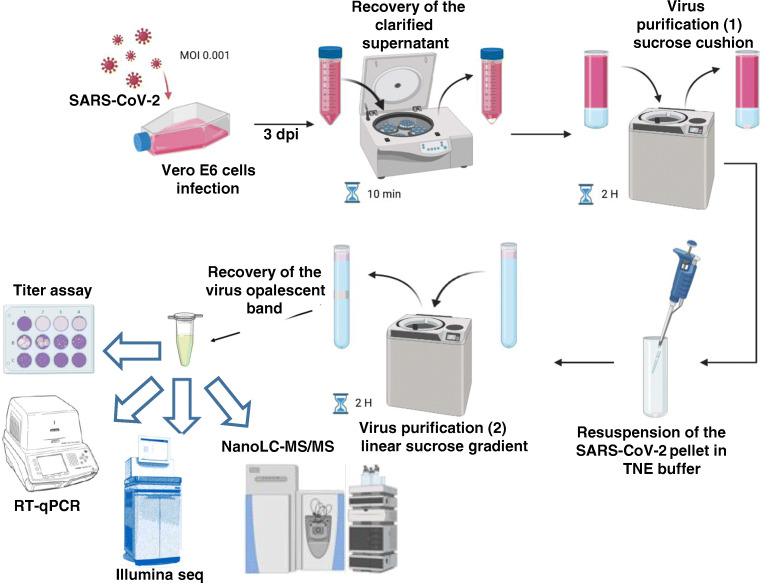
Schematic representation of the experimental design for the production and purification of SARS-CoV-2 viral particles

**Fig. 2:**
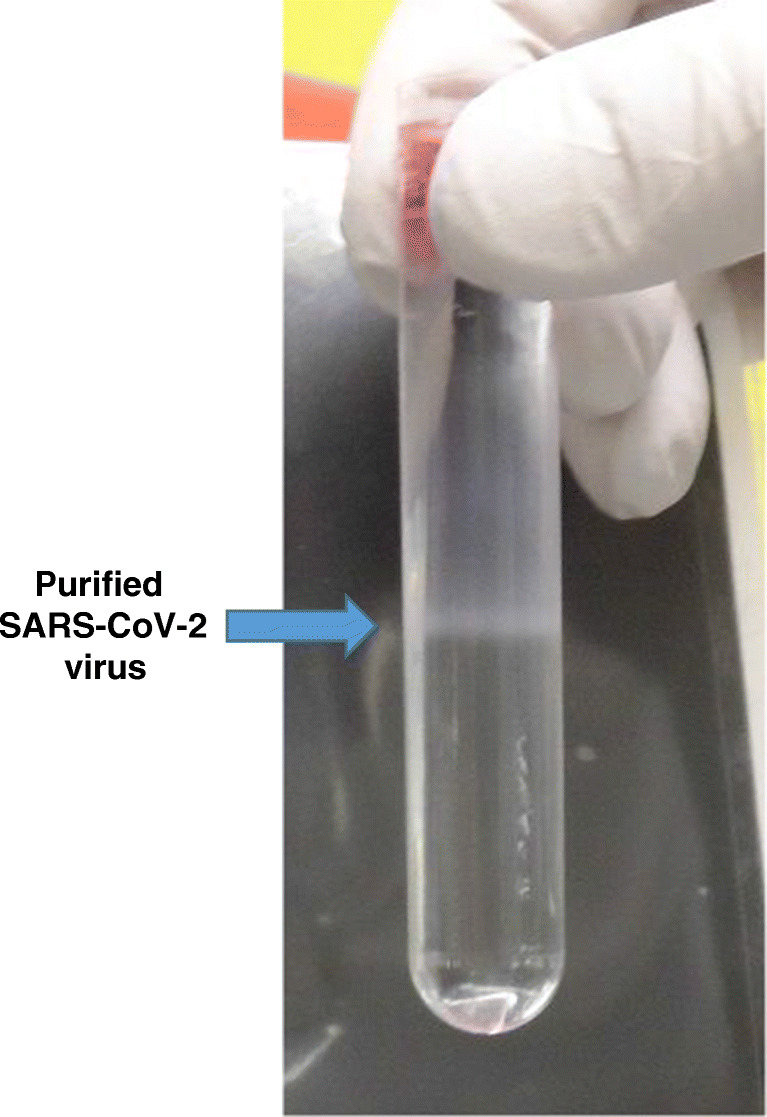
SARS-CoV-2 purification. Virus fraction appearing as a white band following ultracentrifugation on a sucrose gradient

To produce virus particles that could be used as standards in various experiments such as immunogenic-inactivated preparation for antibody production, lateral-flow immunoassay tests, and decontamination assays, we infected Vero E6 cells with the SARS-CoV-2 strain 2019-nCoV/Italy-INMI1 (008N-03893, Genbank MT066156). Figure 1 shows the workflow for the production and purification of viral particles, as well as the analytical methodologies to characterize the purified virus. Seven flasks corresponding to a total of 7 × 10^7^ cells were infected at MOI 0.001 and cultivated for 3 days before harvest. The viral particles were purified via ultracentrifugation on a sucrose cushion first and a sucrose gradient later, and then pooled as a single production batch. While challenging to perform under the BSL-3 conditions required for handling the SARS-CoV-2 virus, this last step of the purification procedure (Fig. 2) is crucial to remove the majority of the contaminant proteins without negatively impacting the concentration of the recovered viral particles. The infectious titre of the purified virus, as estimated by standard plaque assay, was 1.2 × 10^3^ pfu/μL. The viral RNA load estimated by RT-qPCR and the protein concentration of the sample were 2.0 × 10^8^ copies/μL and 0.614 μg/μL, respectively. A total of 1.6 mL of viral particles solution was obtained, thus corresponding to 1.9 × 10^6^ pfu and 3.2 × 10^11^ viral particles. As expected from previous experiments [11], most of the recovered particles were not infectious. A small volume of viral particles used for the mass spectrometry experiments was heat-inactivated. Full inactivation was confirmed by plaque assay.

### Shotgun proteomics of purified SARS-CoV-2 viruses

**Fig. 3:**
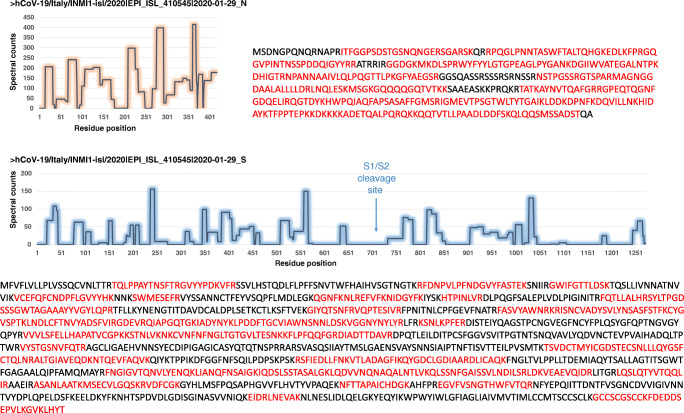
Quantitative peptide mapping on the N and S polypeptides based on spectral counts per residue. SC intensities are indicated per residue position. For this, spectral counts of all peptides covering each residue position were summed. The sequence covered by peptides is indicated in red for each polypeptide

Shotgun proteomics was performed to characterize the purified SARS-CoV-2 viral particles. For this, an aliquot of the sample was subjected to in-gel proteolysis and the resulting peptides were analysed by means of three analytical nanoLC-MS/MS analyses. A total of 107,856 MS/MS spectra were acquired when merging the results of the three analytical replicates. Overall, 68,156 MS/MS spectra were assigned to peptide sequences in the first search round. The resulting assignation rate (63.2%) was in accordance with previously obtained results with similar conditions and datasets [14, 15]. Good reproducibility was obtained for the three analytical runs with 12,512, 12,678, and 12,837 peptide-to-spectrum matches. A total of 11,923 peptide sequences were identified, pointing at the confident detection of 1094 proteins with at least two peptide sequences (see Supplementary Information (ESM) Table S1). A total of 9 SARS-CoV-2 proteins were identified: N (49 peptides, 3318 SC), S (78, 2185), M (11, 531), E (2, 3), Orf1ab-1 (21, 50), Orf9b (6, 28), Orf3a (4, 23), Orf8 (1, 2), and Orf1ab-2 (2, 2). Very good coverage of the sequence of the N (83%) and S (50%) proteins was obtained (Fig. 3). As expected, the number of *Chlorocebus* proteins (928) was higher than those of the *Bos Taurus* contaminants (104), with a total protein abundance of 76.1% versus 7.7%, respectively. The most prominent contaminants in the three analytical runs, as judged by their SC, were the *Chlorocebus* proteins: plectin, heparan sulfate proteoglycan 2, filamin a, myosin heavy chain 9, filamin c, and spectrin alpha. Notwithstanding, their cumulated amounts represented only 8% of the detected proteins. SARS-CoV-2 proteins represented 16.2% of the total. Of note, the dynamic exclusion of 10 s used for recording the data decreases the counts of the most abundant ions. For this reason, viral proteins account probably for a higher percentage. Interestingly, when comparing the profiles of the contaminant proteins obtained by analysing purified viral particles with those resulting from the analysis of infected cell proteome reported previously [11] and acquired under relatively similar conditions, we observed that heparan sulfate proteoglycan 2 (with a fold change ×32), myoferlin (×29), integrin beta (×43), epithelial cell adhesion molecule (×26), versican (×19), glypican (×16), copine (×16), and stress-induced phosphoprotein 1 (×72) were among the most enriched proteins from *Chlorocebus* (see ESM Table S1).

### Alternative interpretation reveals possible non-silent point mutations

**Fig. 4:**
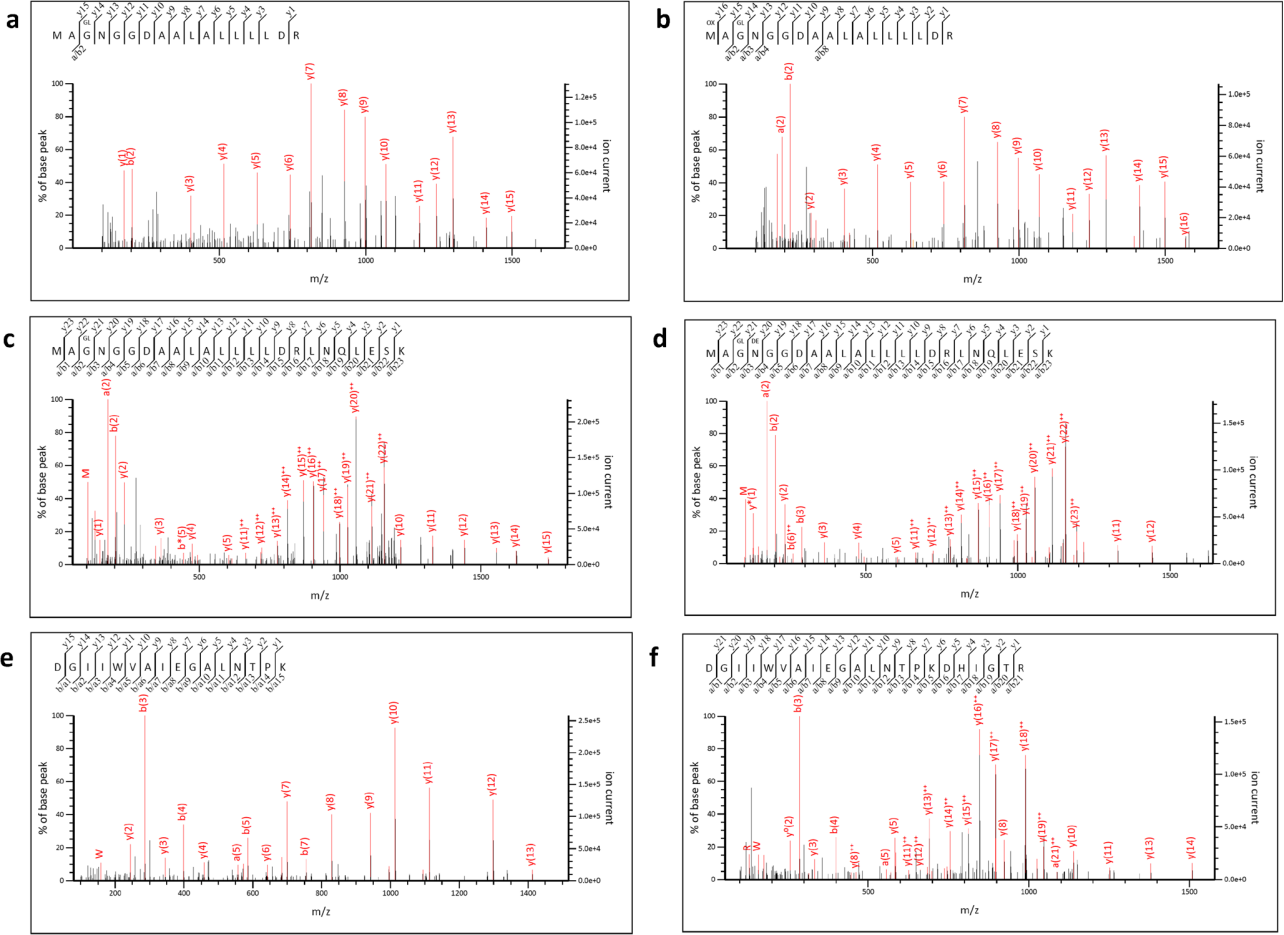
MS/MS spectra highlighting the T135I and G222S mutations of the nucleoprotein N. **a** [210-MA(G→S)NGGDAALALLLLDR-226] variant peptide. **b** [210-MA(G→S)NGGDAALALLLLDR-226] peptide with first residue oxidized. **c** [210-MA(G→S)NGGDAALALLLLDRLNQLESK-233] variant peptide. **d** [210-MA(G→S)NGGDAALALLLLDRLNQLESK-233] peptide with fourth residue deamidated. **e** [128-DGIIWVA(T→I)EGALNTPK-143] variant peptide. **f** [128-DGIIWVA(T→I)EGALNTPKDHIGTR-149] variant peptide

A search of possible non-silent point mutations was performed using the error-tolerant mode of the Mascot software (Proteomic dataset PXD025130). A relatively low number (29) of new MS/MS spectra assignation was obtained. Noteworthy, there were several pieces of evidence pointing at the presence of the same mutation, explained by redundancy due to trypsin miss-cleavage, variable modification, and duplicate spectra (Table 1). The [210-MA(G→S)NGGDAALALLLLDR-226] and [210-MA(G→S)NGGDAALALLLLDRLNQLESK-233] peptides from the N protein were observed with a Gly→Ser modification. Because of the presence of three glycine residues in this sequence, a careful evaluation of the corresponding MS/MS spectra was performed. Figure 4 shows a spectrum of the shorter peptide revealing that the mutation occurred at position 212. Remarkably, the wild-type peptides, [210-MAGNGGDAALALLLLDR-226] and [210-MAGNGGDAALALLLLDRLNQLESK-233], are also detected in the whole dataset with 129 and 169 counts. The two variant peptides [210-MA(G->S)NGGDAALALLLLDR-226] and [210-MA(G->S)NGGDAALALLLLDRLNQLESK-233] were detected with lower counts, 6 and 7, respectively. Therefore, this mutation at position 122 of the N protein appears to occur at less than 5% compared to the wild-type residue.

**Table 1:** Peptide-to-spectrum redundant evidences for G->S variant at position 212 in the nucleoprotein

MS/MS query*	Experimental *m/z*	*z*	Theoretical mass	Error (ppm)	Mis-cleavage	MASCOT score	Start	Peptide sequence	Stop	Modification
21,345	858.952	2	1715.8876	1.0	0	124.42	210	MAGNGGDAALALLLLDR	226	Oxidation (M); Gly->Ser [+30.01]
21,061	850.9534	2	1699.8927	−0.3	0	111.03	210	MAGNGGDAALALLLLDR	226	Gly->Ser [+30.01]
32,209	838.4505	3	2512.3319	−0.9	1	80.39	210	MAGNGGDAALALLLLDRLNQLESK	233	Gly->Ser [+30.01]
32,220	838.7793	3	2513.3159	0.1	1	69.19	210	MAGNGGDAALALLLLDRLNQLESK	233	Deamidated (NQ); Gly->Ser [+30.01]

*MS/MS spectra from the Q26432_MS20-025_Virus-purif_2 raw file

**Fig. 5:**
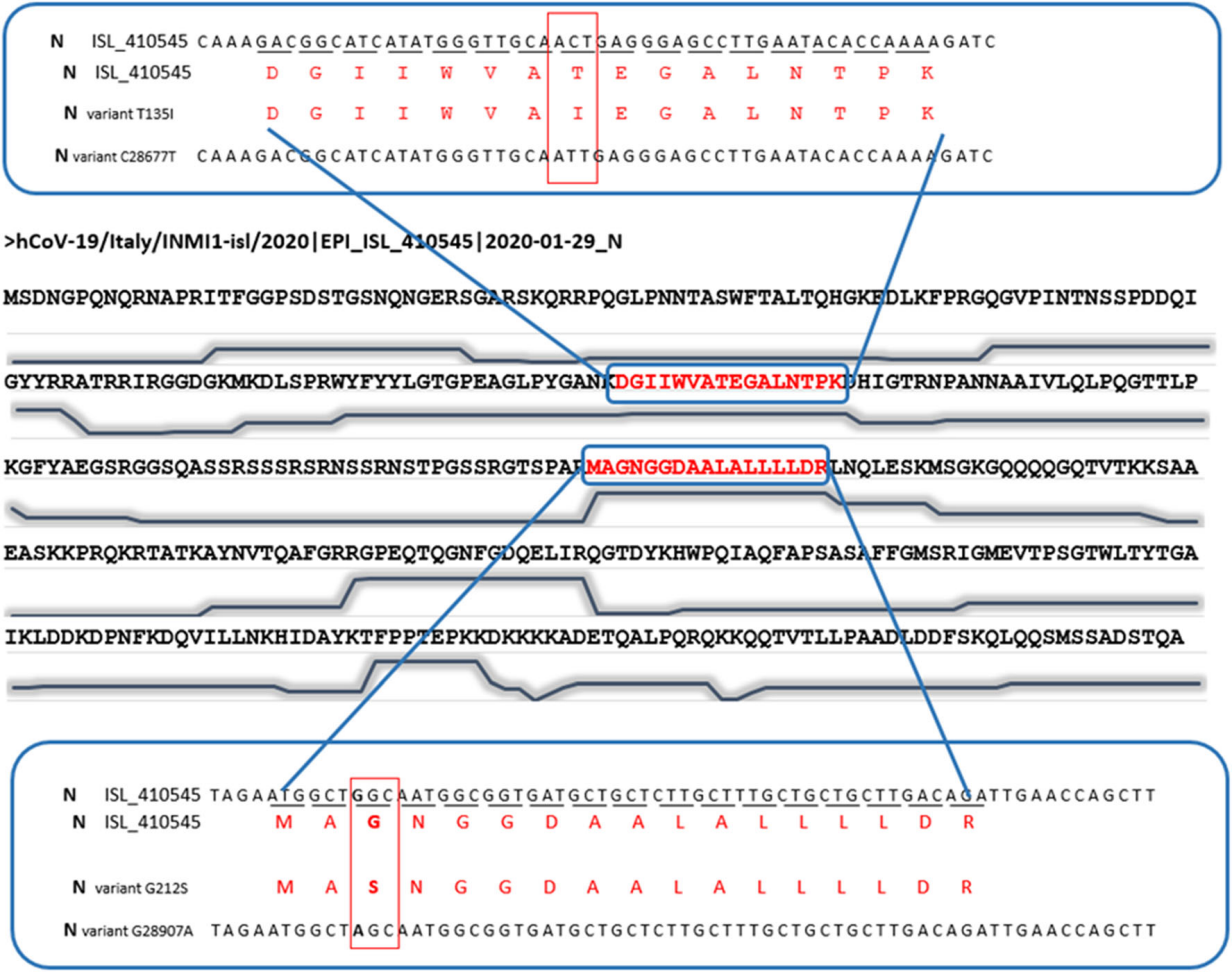
Representation of the nucleoprotein N sequence and the corresponding variant positions at the nucleotide level for the ISL_410545 strain, synchronized on the NC_045512.2 reference genome. The peptide abundances, expressed as spectral counts per residue, are schematized under the corresponding amino acid polypeptide regions

Another search was performed using a database compiling all the known variant sequences based on the SARS-CoV-2 genomes deposited in GISAID (Proteomic dataset PXD025131). Two additional variant peptides, [128-DGIIWVA(T→I)EGALNTPK-143] and [128-DGIIWVA(T→I)EGALNTPKDHIGTR-149], were detected which collectively point at a threonine changed into an isoleucine residue at position 135 in the N protein. This modification was observed in two variants reported in GISAID: hCoV-19/USA/WI-30/2020|EPI_ISL_421288 and hCoV-19/India/NCDC-02252/2020|EPI_ISL_435097. In this case, the mutated peptides were detected with lower counts, 14 and 6, respectively, than the wild-type peptides, 234 and 267. Therefore, this mutation at position 135 in the N protein appears to occur at less than 4% compared to the wild-type residue. Figure 5 shows the nucleoprotein sequence with the two detected mutations at the polypeptide sequence level, as well as the nucleotide mutations that could generate these changes. A transversion (Ggc/Agc) in the N gene resulted in the mutation of glycine at position 212 into a serine. This transversion was not reported in the GISAID database. Another single nucleotide transversion (aCt/aTt) could explain the variant observed at position 135. As it was detected in the genomes of several variants, it occurs in a permissive region of the protein. The whole genome sequence confirmed the presence of RNA molecules encompassing the two transversions as shown in Table 2. C and T nucleotides were detected at position 28,677; the mutated form was detected in 1.9% of the high-quality reads. G and A nucleotides were detected at position 28,907; the mutated nucleotide accounted for 0.8% of the reads. Although these mutations were in minor amounts compared to the wild-type nucleotides, they were easily distinguished from the other possible base calls.

**Table 2:** Whole SARS-CoV-2 genome sequence confirms the presence of mutated RNA molecules

Nucleotide position	Number of reads				Percentage of reads			
	A	C	G	T	A	C	G	T
28,676	426,923	58	590	57	99.9	0.0	0.1	0.0
**28,677**	24	**422,337**	15	**8345**	0.0	**98.1**	0.0	**1.9**
28,678	39	345	165	430,540	0.0	0.1	0.0	99.9
28,906	104	520	58	503,783	0.0	0.1	0.0	99.9
**28,907**	**4175**	11	**494,689**	42	**0.8**	0.0	**99.2**	0.0
28,908	214	12	496,368	55	0.1	0.0	99.9	0.0

Only reads with high quality (>Q30) were taken into account. Only the key nucleotide positions and their surrounding nucleotides for the ISL_410545 strain, synchronized on the NC_045512.2 reference genome, are indicated. Bold letters indicate the two positions of interest at 28,677 and 28,907 and highlight the two possible nucleotides

## Discussion

In the present study, we applied a mass spectrometry-based approach to profile SARS-CoV-2 virions purified following infection of Vero E6 cells. We showed that shotgun proteomics not only allows a comprehensive proteomic description of the subset of host proteins potentially associated with intact virions but highlights the heterogeneity of structural proteins produced by infected cells in vitro.

The proteomic profile produced by LC-MS/MS and its comparison with the previously obtained proteome of infected Vero E6 cells revealed an enrichment of *Chlorocebus* proteins such as heparan sulfate proteoglycan 2, myoferlin, integrin beta, epithelial cell adhesion molecule, versican, glypican, copine, and stress-induced phosphoprotein 1 among the purified virions. Heparan sulfate proteoglycan 2 is a cell-surface endocytosis receptor involved in diverse macromolecular cargo and its role in SARS-CoV-2 cell attachment has been established recently [16]. Myoferlin is a multifunctional protein that was involved in the invasion of tumour cells [17]. Integrin beta is also a cell surface receptor and its role as putative host cell entry was discussed [18]. Versican is a key component of the extracellular matrix involved in cell adhesion, proliferation, and migration. Glypican is a protein anchored to the cell membrane involved in developmental morphogenesis. Copine is a calcium-dependent membrane protein with a role in membrane trafficking. Stress-induced phosphoprotein 1 shows some domain similarities with a mitochondrial import receptor, and thus could be a possible membrane receptor. All these proteins may be enriched in the purified sample due to their strong affinity with SARS-CoV-2 viral particles, potentially via the Spike protein. As exemplified with heparan sulfate proteoglycan 2 (16), these proteins could be involved in SARS-CoV-2 infectious mechanisms and their respective roles are probably worth being further investigated.

We identified by tandem mass spectrometry the presence of variants in low but detectable quantities in the purified SARS-CoV-2 viral particles and particularly in the N protein. At the early stage of the pandemic, the SARS-CoV-2 genome was considered as relatively stable and mainly evolving under the effect of point mutations generally neutral and without impact on virulence and severity [19]. Analysis of SARS-CoV-2 proteome sequences deposited in the GISAID database over the past year showed that SARS-CoV-2 proteins are mutating at substantially different rates, with most of the viral proteins exhibiting little mutational variability while the Spike and Nucleocapsid proteins have the highest one [20]. The emergence of new variants of SARS-CoV-2 called “variants of concern” triggered a new phase of the pandemic [5]. Indeed, the virus is currently subjected to a higher pressure than before as it now actively explores its evolutionary space while confronting in higher numbers hosts with an efficient immune response compared to the first versions of SARS-CoV-2 (either infected patients that suffered COVID-19 in the earlier stages of the pandemic or vaccinated subjects). While a vast majority of vaccines are developed for targeting highly conserved regions of the S protein or parts thereof, the N protein is also envisioned for vaccine development [21]. N-Protein variants such as those deciphered in the present study might be important for such developments. While the vaccine evading problem may be a Black Swan theoretical idea [22], understanding how SARS-CoV-2 evolves and anticipating the response to these novel variants remain of utmost importance [23]. A very recent study based on whole genome SARS-CoV-2 deep sequencing of numerous clinical samples reported low levels of within-host diversity, but a dynamic landscape during infection [24].

The proteomics data indicated that 16% of spectral counts were assigned to viral proteins in the purified fraction, but the dynamic exclusion used for selecting peptide for fragmentation decreases the counts of the most abundant proteins, therefore minoring the viral protein ratio by an important factor. With the protein concentration of the sample being established at 0.614 μg/μL, at least 10 ng/μL of viral proteins is present. The structure of the viral particle revealed the presence of 26 (±15) copies of the S protein and 26 (±11) copies of the ribonucleoprotein N per virion [1]. Thus, the protein content of a SARS-CoV-2 viral particle should have an average molecular weight of ≈5.5 MDa. The sample content in SARS-CoV-2 particles is estimated to be 11 × 10^8^ copies/μL while the viral RNA load was measured by RT-qPCR to reach 2.0 × 10^8^ copies/μL; thus, at least one-fifth of the viral proteins could be assembled around a RNA molecule.

It was recently estimated that each infected person could carry 10^9^–10^11^ virions during peak infection, but only a fraction of these particles are infective as the 50% tissue culture infective dose (TCID_50_) was estimated to be 10,000 lower, i.e. 10^5^–10^7^. Here, our purified SARS-CoV-2 viral particles were even less infective (167,000 lower). The total number of virions produced throughout an infection was estimated to be ten times the peak infection load. Therefore, each infected patient could produce 10^10^–10^12^ virions; and thus, a great number of variants could be theoretically produced by a single person during their infection as suggested by our experimental results. The latest epidemiological reports mentioned that SARS-CoV-2 has infected over 132 million people, thus multiplying the possibilities of creating new variants by a huge factor. Intra-host genomic plasticity has been reported after genome sequencing of samples from COVID-19 patients. Karamitros et al. [25] described the occurrence of tens of low or higher frequency single nucleotide variations in several viral proteins. This intra-host diversity of SARS-CoV-2 was also found to be host-to-host transmitted [26]. Indeed, SARS-CoV-2 viruses inhabit the host as a population of variants (the so-called quasispecies) and this diversity changes over time. Based on metatranscriptomics, a recent study revealed the co-existence of different genotypes within the same patient [27]. Finally, recent works have shown that in some cells mutations on the spike protein could lead to loss of furin cleavage and become dominant, while wild-type SARS-CoV-2 sequence is maintained at a low percentage [28]. Shotgun proteomics and mass spectrometry could be useful for quickly assessing these variants as the methodology does not rely on amplification of the molecules that could introduce some bias.

Information regarding the SARS-CoV-2 peptide landscape is of crucial interest for mass spectrometry-based diagnostic. Peptide targets were first proposed based on their mass spectrometry detectability observed by shotgun proteomics on preliminary datasets as well as on their degree of conservation among known variants [29]. Over the last months, several studies have shown how mass spectrometry could be implemented for SARS-CoV-2 diagnosis from gargle samples or nasopharyngeal swabs [30]. Interestingly, these acquisition methods could be quickly adapted to detect new variants as they do not require specific reagents. While the mutations have been identified here from viral particles purified from a cell culture preparation, it is now of utmost interest to test whether mass spectrometry can be used to evaluate SARS-CoV-2 evolution in clinical samples.

In conclusion, the present study shows how shotgun proteomics can be used in routine experiments to easily check for the homogeneity of viral particles preparations. We confirmed that the production of SARS-CoV-2 viruses in the Vero E6 cell line induced the appearance of mutated structural proteins. While top-down analysis of these polypeptides could be of high interest to determine if various mutations could be encompassed per polypeptide, shotgun proteomics allows to quickly identify the positions that are prone to mutations without specific constraints. Clearly, such in vitro experiment indicated that new variants not yet catalogued in GISAID may appear. While genome sequencing of the SARS-CoV-2 genome is still quite tedious depending on the viral charge of the sample, mass spectrometry could be an alternative solution to quickly assess the mutational landscape of any samples. Among the possible applications, this methodology could be helpful to monitor the virus evolution from immunocompromised patients suffering from COVID-19 and subjected to long-term therapy.

## Supplementary Information

ESM 1 (XLSX 94 kb)

## Data Availability

The mass spectrometry and proteomics datasets are available through the ProteomeXchange Consortium via the PRIDE partner repository (https://www.ebi.ac.uk/pride/), under dataset identifiers PXD025131 and 10.6019/PXD025131 for the direct interpretation and PXD025130 and 10.6019/PXD025130 for the error-tolerant interpretation.
